# Understanding individual and collective response to climate change: The role of a self-other mismatch

**DOI:** 10.3389/fpsyg.2022.935209

**Published:** 2022-09-29

**Authors:** Rosie Harrington, Armelle Nugier, Kamilla Khamzina, Serge Guimond, Sophie Monceau, Michel Streith

**Affiliations:** ^1^LAPSCO (Laboratoire de Psychologie Sociale et Cognitive), Université Clermont Auvergne et CNRS, Clermont-Ferrand, France; ^2^PSITEC-ULR 4072, Psychologie: Interactions, Temps, Emotions, Cognition, Lille, France

**Keywords:** norms, mismatch, veganism, behavioral intentions, collective engagement

## Abstract

Several scientists have shown the importance of mitigating global warming and have highlighted a need for major social change, particularly when it comes to meat consumption and collective engagement. In the present study (*N* = 486), we conducted a cross-sectional study to test the mismatch model, which aims at explaining what motivates individuals to participate in normative change. This model stipulates that perceiving a self—other difference in pro-environmental attitudes is the starting point and can motivate people to have high pro-environmental intentions. This mismatch effect is explained by participants’ willingness to participate in normative and social change: people that perceive a gap between their personal attitude and the social norm should be more willing to participate in normative change. This should then motivate them to have high pro-environmental intentions on an individual and group level. The results confirm the hypothesized model on an individual and group level and explain how people can be motivated to participate in normative change. Implications of these findings and the need for further studies are discussed.

## Introduction

The intergovernmental panel on climate change’s (IPCC, 2021) sixth Assessment Report explains that “human influence has warmed the climate at a rate that is unprecedented in at least the last 2000 years” and that climate change has already had a visible impact on the average weather and climate conditions in every part of the world. For example, the number of heat waves, droughts, and tropical cyclones caused by human influence has increased since the fifth assessment report that was published only 7 years ago. In this report, IPCC also simulated different possibilities for what is to come and their results show that even if the optimistic option plays out (for which we would have to drastically reduce our CO_2_ and other greenhouse gas emissions), the global surface temperature will continue to rise until the mid-century mark. As the global temperature rises, these extreme meteorological conditions (heat waves, droughts, etc.) will occur more frequently and intensely.

Combined with other similar studies relating the negative impact of human-induced climate change on accessible resources (Watson et al., 1998; Christensen et al., 2004) and biodiversity (Willis and Bhagwat, 2009; Bellard et al., 2012), these results show how important it is to change how we interact with our environment. Indeed, mitigating the effects of climate change could somewhat help to avoid the disastrous effects of this phenomenon on our day-to-day lives. Different actions are possible to participate in the mitigation of climate change with some of them being more efficient and easily accessible than others. According to Wynes and Nicholas (2017), for example, following a plant-based diet (i.e., being vegetarian or vegan) is one of the most impactful individual behaviours in terms of climate change mitigation, with having one less child or using renewable energies (see also Barma et al., 2017), and public transport or walking (see also Fuglestvedt et al., 2008), compared to other repeated behaviours like recycling, washing clothes in cold water or even using led lightbulbs. Aside from acting on an individual level, people can also act on a societal level. Indeed, they can vote for green party politicians, organise, and/or participate in protests calling for more governmental action (see Aldy et al., 2001; Adger, 2003; Fritsche et al., 2011). Of importance, and according to Wullenkord and Hamann (2021), see also (Schulte et al., 2021), acting on an individual and group level is complementary and equally important for overcoming the effects of climate change.

Even if these different possibilities exist, and society acknowledges them (see Steg, 2018), a lot of people—including climate change deniers as well as those who are more aware of climate change—still do not necessarily behave pro-environmentally (Whitmarsh and Capstick, 2018). Thus, part of climate change mitigation has now started to include research on what motivates these behaviours and how social change can occur. In this article, we empirically test the mismatch model (Khamzina et al., 2021; Deffuant et al., 2022) to explain what can motivate individuals to act pro-environmentally on a group and individual level, and participate in normative change.

## The mismatch effect on pro-environmental intentions

Social psychology is one of the many fields that offer explanations for why people act in various ways and throughout the different theories and studies, two behavioural predictors seem essential to understanding pro-environmental action: social norms and attitudes (Ajzen and Fishbein, 1977; Ajzen, 1991). Attitudes are generally considered to be an evaluation of a certain object, concept, or person, which ranges from extremely negative to extremely positive (Bohner and Dickel, 2011). Ajzen (1991) and Ajzen et al. (2018) then offers a more detailed definition with two main components: an affective dimension that focuses on what an individual personally believes about the behaviour, and a cognitive dimension that is more about what we think the consequences of said behaviour are. Researchers secondly define social norms as a ‘collective awareness about the preferred, and appropriate behaviours among a certain group of people’ (Chung and Rimal, 2016). Different theories then explain that different types of norms exist. For example, Cialdini et al. (1990) theory offer two types of norms: descriptive norms and injunctive norms. Descriptive norms represent how people in a specific group actually behave, and injunctive norms are standards that members of the group are expected to follow and expect others to follow in a given social situation. These norms indicate whether behaviour is approved of or frowned upon, whereas descriptive norms concern what people are actually doing.

Multiple studies have shown that attitudes and social norms play an important role in predicting intentions to act pro-environmentally (Fife-Schaw et al., 2007; Krispenz and Bertrams, 2020; Niemiec et al., 2020). Most of these studies and the underlying theories consider attitudes and social norms to be independent concepts, with independent effects on intentions and behaviour (Bagozzi and Schnedlitz, 1985; Ajzen, 1988, 1991). Nevertheless, the few studies that have tested the interaction effect of attitudes and norms found that it significantly affects the participants’ intentions to engage in a particular behaviour (Grube and Morgan, 1990; Prentice and Miller, 1993; Fife-Schaw et al., 2007), notably when the two variables are mismatched—when one is in favour of the behaviour and the other one is against. Researchers first hypothesized that when an individual disagrees with public opinion, they would rather conform to what others believe rather than go against said social norm (Asch, 1951; Noelle-Neumann, 1974, 1993). Thus, people would tend to change their behaviour to fit with those of others (i.e., a normative influence hypothesis; Asch, 1951; Acock and DeFleur, 1972; Grube and Morgan, 1990). Studies testing Noelle-Neumann’s spiral of silence theory have, however, mixed results and often find a weak correlation between majority support and the expression of personal opinions (Scheufele and Moy, 2000; Katz and Fialkoff, 2017). The mixed results could be due to the theory not applying to all situations. For example, it does not explain how and why societies evolve and change over time: normative change cannot happen if people always conform to the social norm. Therefore, some researchers have hypothesized that people sometimes share their dissident opinion and that minorities can significantly influence opinions (Moscovici et al., 1969; Moscovici, 1991). Others have also suggested that observing a difference between one’s personal attitude and perceived social norms is the starting point and can make us stand more strongly in our position and not conform to social norms (Khamzina et al., 2021; Deffuant et al., 2022).

Khamzina et al. (2021), see also Deffuant et al. (2022) call this interaction effect and its predictions “the mismatch hypothesis.” The first central idea in this hypothesis is that the mismatch between perceived social norms and personal attitudes can be a source of motivation to have high intentions to act pro-environmentally—notably when the individual perceived their personal attitude to be in favour of the behaviour but not the social norm. In a series of studies, Khamzina et al. (2021) effectively confirm this hypothesis: intentions to convert to organic farming were significantly higher when the farmers’ attitudes and perceived social norms were mismatched (with the personal attitude being in favour and perceived norms being against), compared to the other possibilities. This mismatch effect is not simply people ignoring the social norm because they do not identify with the social group: people who identify as a member of the said group can also be motivated to act against social norms (Packer, 2009; Packer and Chasteen, 2010). These studies stem from the normative conflict model that states that ‘strongly identified members are attentive to group-related problems and perceptions that the status quo is harmful to the collective may trigger expression of dissenting opinions’ (Packer, 2008, pp.1). On this basis, a second central part of this mismatch model theorised by Khamzina et al. (2021) is that the mismatch effect is rooted in wanting to change the group for the better and pushing for social change. Khamzina and collaborators, therefore, suggest, although do not test, that the mismatch effect on intentions can be explained by a willingness to change social norms. They suggest that when individuals perceive a mismatch between the social norm and their own personal attitude, they will want to change this social norm to make better the group, and this should result in more pro-environmental behaviours. This willingness to change norms would then push people to have a level of pro-environmental intentions that coincides with their own personal attitude.

## Study overview

The main goal of this article is to investigate the theorized mismatch model and the key component of willingness to change social norms that, according to our knowledge, has yet to be empirically tested. We built our study on a specific environmental challenge: meat consumption. Given that meat consumption explains a significant part of greenhouse gas emissions due to human activity (Tukker and Jansen, 2006; Aubin, 2014), reducing our dietary intake of meat would contribute considerably to the fight against climate change (Salonen and Helne, 2012; Chai et al., 2019). In France and most western societies, however, diets with low or no meat intake are rare (Sanchez-Sabate and Sabaté, 2019) and there is often a strong national fightback when it is the case (Cholez, 2021; Schittly, 2021). People acting pro-environmentally, in this sense, are part of a minority in France. Hence, this is an area in which social change is crucial from a climate change mitigation perspective. As the mismatch model might explain individual behaviour in times of social change, vegetarians’ behaviour could, therefore, be explained by the mismatch model. Indeed, previous literature shows that being part of a minority can make minority members feel different and distant from other members of the group (Hassouneh et al., 2014; Gutmann-Kahn and Lindstrom, 2015), whether it stems from having a different identity or even believing to have diverging opinions from majority members. Even if being vegetarian or vegan is not quite the same as other marginalized groups because they are a minority group based on choice not by biological trait, research shows that similar processes of distance and stigmatization still occur (Bresnahan et al., 2016; Markowski and Roxburgh, 2019). For example, vegetarianism is often treated as a deviant practice that requires explanation (Wilson et al., 2004; de Groeve et al., 2021). For these reasons, vegetarians could perceive a bigger self—other difference than non-vegetarians, and this could then activate the mismatch pattern. In the present study, we, therefore, compare vegetarians and vegans (i.e., veg*ns) to people who still eat meat to see if it is effectively the case.

We first hypothesized that veg*ns would feel more strongly in mismatch, with their personal attitude higher than the social norms, compared to participants who still eat meat regularly (H1). We also predict that veg*ns will have higher pro-environmental intentions than non-veg*ns, at an individual level (H2a). While studies on non-conformism have mainly looked at individual action, we also wanted to extend these results to group-level action: as vegetarians are more highly in mismatch, we predict veg*ns should also have higher group level intentions than non-veg*ns (H2b). Finally, to provide a test of the mechanism underlying the impact of mismatch on pro-environmental intentions, we hypothesized that the relation between mismatch and pro-environmental intentions will be mediated by the willingness of participants to change social norms. More precisely, we tested a serial mediation model in which veg*ns participants should be more highly in mismatch, which then heightens their willingness to change norms. The latter then increases intentions to behave pro-environmentally at an individual level (H3a) and group level (H3b).

## Materials and methods

### Participants

The final sample comprised 486 volunteers (97,3% French native; 78% women, 21 % men, 1% others) who ranged from 18 to 76 years (*M*_age_ = 34.84, *SD* = 12.80). Most of the participants had a university education (96,5%). Two hundred forty participants (49,4 %) declared themselves as vegetarian or vegan and 246 (50,6%) still eat meat (see Supplementary materials for additional information). This online study was conducted from 26 July to 9 September 2021.

### Procedure

We approached potential participants on a variety of social network groups and proceeded through snowball sampling. As the aim of the study was to compare two sub-groups of the French population that either act pro-environmentally (vegans) or do not (omnivores), we reached out to as many veg*n and non-veg*n groups as possible. Consequently, we had well-balanced groups, which was ideal for the planned statistical analysis. The study was presented as an online study on global warming and meat consumption. After briefing about the purpose of the study, participants answered the questionnaire and were then, debriefed and thanked.

### Measures

All answers were given on 9-point Likert scales, ranging from (1) “*strongly disagree*” to (9) “*strongly agree*.” Participants’ composite scores for each measure were averaged.

*Attitudes* toward eating less meat in order to reduce global warming were measured using four statements inspired by Wan et al. (2017). We adapted their questions on attitudes toward recycling to our target behaviour (changed “recycling is rewarding” to “eating less meat is rewarding” for example), α = 0.88, *M* = 7.24, *SD* = 2.05. Perceived *social norms* in France were measured using the Guimond et al. (2013, 2015) method where we replace “I think that” of each item that measured attitudes with “Most French people think that” (e.g., “Most French people think that eating less meat is a good idea in order to reduce global warming”), α = 0.90, *M* = 4.44, *SD* = 2.00. The two measures were counterbalanced (see Guimond et al., 2013, 2015). A mismatch index was created by subtracting ratings of social norms from ratings of attitudes. A high score on this measure indicates a level of mismatch in favour of personal attitudes. Participants’ *willingness to participate in normative change* was measured with one statement (e.g., “I would like to participate in the changing of the meat-consumption norm in France”), *M* = 7.31, *SD* = 2.25. *Individual-level intention* to act against global warming by eating less meat was assessed with four statements (e.g., “To fight against climate change, as an alternative to meat products, I intend to eat more legumes, cereals, or plant-based proteins) (e.g., tofu, red beans, lentils, chickpeas, etc.”), α = 0.83, *M* = 7.43, *SD* = 1.92. *Group-level intention* to act against global warming was assessed with two statements (e.g., “During the next presidential elections, I intend to vote for a political party, whose program would be very protective of the environment”; “I plan to participate in upcoming regional or national climate events”), *r* = 0.41, *p* < 0.001, *M* = 5.33, *SD* = 2.25. These two levels of intentions are positively correlated (*r* = 0.47, *p* < 0.001).

### Demographic and dispositional variables

Participants answered socio-demographic questions relating to their gender, age, country of birth, country of residence, education level, diet (ie., veg*ns or not), political orientation [assessed by indicating their position on a scale ranging from (1) extreme left-wing to (9) extreme right-wing, *M* = 3.94, *SD* = 1.59], identification as a national (French) citizen [assessed by indicating their position on a scale ranging from (1) “*not at all*” to (9) “*extremely*”, *M* = 6.58, *SD* = 2.05], and the perception of themselves as an environmental activist [assessed by indicating their position on a scale ranging from (1) “*not at all*” to (9) “*extremely*,” *M* = 4.38, *SD* = 1.67]. Supplementary analyses controlling these factors were conducted and the results remained unchanged.

## Results

### Attitudes and perceived group norm: A significant mismatch

**FIGURE 1:**
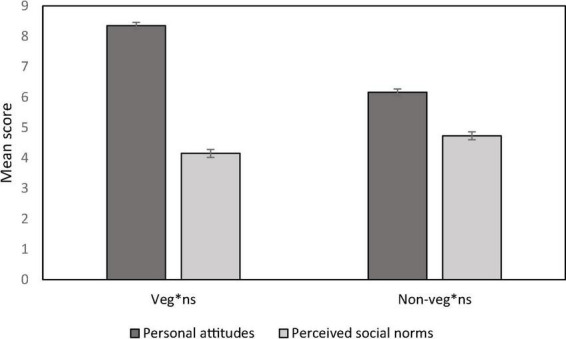
Veg*ns and non-veg*ns’ mean scores of personal attitudes and perceived norms toward the reduction of meat consumption as a mean to reduce global warming. The bars are standard error bars.

We conducted a 2 (Diet: veg*ns vs. non-veg*ns) * 2 (Type of measure: personal attitude vs. social norm) mixed ANOVA with the last variable as a within-participant factor. All reported effects are significant at *p* < 0.001 except where it is mentioned. We observed a main effect of type of measures [*F*(1, 484) = 713.37, η^2^_p_ = 0.59], yielding a significant difference between personal attitudes and perceived social norms. Overall, participants had a more favourable attitude toward the reduction of meat consumption as a means to fight against global warming (*M* = 7.24, *SD* = 2.05) than what they perceived as the social norm in France (*M* = 4.44, *SD* = 2.00). As predicted, this main effect was qualified by a significant interaction with the participants diet [*F*(1, 484) = 171.79, η^2^_p_ = 0.26, see Figure 1]. Veg*n participants were more positive toward the reduction of meat consumption as a mean to fight against global warming (*M* = 8.35, *SE* = 0.11) than non-veg*n participants [*M* = 6.16, *SE* = 0.11, *F*(1, 484) = 194.90, η^2^p = 0.29]. They perceived slightly less (*M* = 4.15, *SE* = 0.13) than non-veg*n participants (*M* = 4.73, *SE* = 0.12) that French people are favourable toward the reduction of meat consumption as a mean to fight against global warming [*F*(1, 484) = 10.36, η^2^p = 0.02]. Of interest, the perceived difference between attitude and social norms was greater for participants that follow a veg*n diet [mean difference = 4.21, *SE* = 0.15; *F*(1, 484) = 782.99, η^2^_p_ = 0.62] compared with participants who still eat meat [mean difference = 1.44, *SE* = 0.15; *F*(1, 484) = 93.66, η^2^_p_ = 0.16]. Thus, H1 was confirmed: veg*ns of our sample were more strongly in mismatch, with their attitude higher than the perceived social norms, compared to participants who still eat meat regularly.

### Testing our model: Mediations on individual and group pro-environmental intentions

**FIGURE 2:**
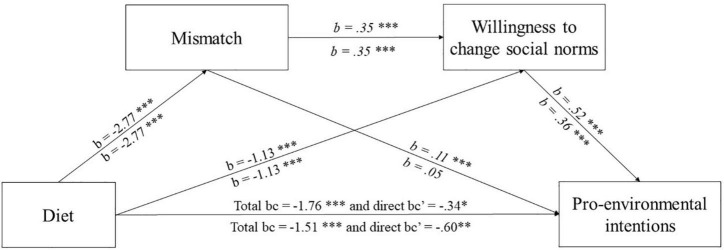
Mediation model A (above the arrows) and model B (under the arrows). They were assessed using Hayes process model 6, evaluating through mismatch perception and willingness to engage in normative change as mediators of the relationship between the diet that participants followed (coded: 0 = Veg*ns; 1 = non-veg*ns); and the intentions to act against the climate change at an individual-level and the diet that participants followed and the intentions to act against the climate change at a group-level, respectively. b = unstandardized coefficients. *p < 0.05; **p < 0.01; ***p < 0.001.

To test whether participants’ diet indirectly influenced their tendencies to act pro-environmentally at an individual (Model A) and group level (Model B) through causally linked multiple mediators of mismatch and willingness to participate in normative change, two serial mediation analyses (Model 6 in PROCESS, 5000 percentile bootstrap) were conducted with the bootstrap method (Hayes, 2014). The paths for the full process model are shown in Figure 2 and their corresponding coefficients and 95% Cis are shown in Tables 1, 2 (see Supplementary materials for an extended description). Figure 2 shows the serial mediations.

**TABLE 1:** Direct, indirect, and total effects of the hypothesized model A.

Model pathways	*b*	SE	*t*	*p*	LL95%CI	UL95%CI
***Direct and total effects***						
Diet → Mismatch	–2.77	0.21	–13.10	0.001	–3.19	–2.36
Diet → Willingness	–1.13	0.19	–5.86	0.001	–1.50	–0.75
Mismatch → Willingness	0.35	0.03	9.81	0.001	0.28	0.42
Mismatch → Individual level intention	0.11	0.02	4.42	0.001	0.06	0.17
Willingness → Individual level intention	0.52	0.03	16.95	0.001	0.46	0.58
Total model effect	–1.76	0.15	–11.38	0.001	–2.07	–1.46
Direct effect	–0.34	0.13	–2.55	0.011	–0.61	–0.08

**Indirect effects**	**Effect**	**SE**			**LL95%CI**	**UL95%CI**

Total	–1.42	0.14			–1.70	–1.14
Diet → Mismatch → Individual level intention	–0.32	0.08			–0.49	–0.17
Diet → Willingness → Individual level intention	–0.59	0.10			–0.80	–0.39
Diet → Mismatch → Willingness → Individual level intention	–0.50	0.08			–0.67	–0.36

***b*** = unstandardized coefficients.

**TABLE 2:** Direct, indirect, and total effects of the hypothesized model B.

Model pathways	*b*	SE	*t*	*p*	LL 95%CI	UL 95%CI
**Direct and total effects**						
Diet → Mismatch	–2.77	0.21	–13.10	0.001	–3.19	–2.36
Diet → Willingness	–1.13	0.19	–5.86	0.001	–1.50	–0.75
Mismatch → Willingness	0.35	0.03	9.81	0.001	0.28	0.42
Mismatch → Group level intention	0.05	0.04	1.22	0.224	–0.03	0.13
Willingness → Group level intention	0.36	0.05	7.41	0.001	0.26	0.46
Total model effect	–1.51	0.19	–7.80	0.001	–1.89	–1.13
Direct effect	–0.60	0.21	–2.81	0.005	–1.03	–0.18

**Indirect effects**	**Effect**	**SE**			**LL95%CI**	**UL95%CI**

Total	–0.90	0.13			–1.17	–0.65
Diet → Mismatch → Group level intention	–0.14	0.12			–0.38	0.10
Diet → Willingness → Group level intention	–0.41	0.08			–0.59	–0.26
Diet → Mismatch → Willingness → Group level intention	–0.35	0.06			–0.49	–0.23

***b*** = unstandardized coefficients.

As predicted, it was found that participants’ diet significantly predicted the mismatch perception (*b* = –2.77, 95%CI [–3.19; –2.36]). Participants’ diet (*b* = –1.13, 95%CI [–1.50; –0.75]) and mismatch perception (*b* = 0.35, 95%CI [0.28; 0.42]) also significantly predicted the willingness to participate in normative change. For the Model A and Model B, both the total effect of diet on intention to act at an individual or a group level against climate change (Model A: *b* = –1.76, 95%CI [–2.07; –1.46]*; R^2^* = 0.21*;* Model B: *b* = –1.51, 95%CI [–1.89; –1.13], *R*^2^ = 0.11) and the total direct effects when controlling for the mediators were significant (Model A: *b* = –0.34, 95%CI [–0.61; –0.08]*;* and Model B*: b* = –0.60, 95%CI [–1.03; –0.18]^1^).

Of importance, H3a and H3b were supported. For both Model A and Model B, the total indirect effects were significant (Model A: *effect* = –1.42, 95%CI [–1.70; –1.14], and Model B: *effect* = –0.90, 95%CI [–1.17; –0.65]), with a significant serial mediation effect being observed from participant’s diet *via* mismatch perception and willingness to participate in normative change in intention to act at an individual level (Model A: *effect* = –0.50, 95%CI [–0.67; –0.36]) and to intention to act against climate change at a group level (Model B: *effect* = –0.35, 95%CI [–0.49; –0.23]). The specific indirect effect through mismatch only was significant for Model A (*effect* = –0.32, 95%CI [–0.49; –0.17]) but not for Model B (*effect* = –0.14, 95%CI [–0.38;.10]) whereas the specific indirect effect through willingness to participate in normative change only was significant for both Model A (*effect* = –0.59, 95%CI [–0.80; –0.39]) and model B (*effect* = –0.41, 95%CI [–0.59; -0.26]).

Overall, these findings indicate that veg*n participants have a strong intention to behave pro-environmentally at both individual level (i.e., eating no meat, H3a) and group level (i.e., voting for a green political party or participating in climate events, H3b), because the perceived mismatch between their attitude and social norms is associated with their willingness to change social norms toward meat consumption.

## Discussion

In this study, we investigated the mismatch model by applying it to a minority that already acts pro-environmentally (i.e., veg*ns). We compared vegetarians and vegans to omnivores to see if the mismatch between personal attitudes and perceived social norms can explain how a minority maintains its high pro-environmental intentions on an individual level. Our second goal was to see if this same model can also motivate other levels of pro-environmental intentions (i.e., on a group level). When testing the model on pro-environmental intentions on an individual and group level, each path of the mediation is significant and confirmed our expectations—even when controlling for demographic and dispositional variables. First, vegetarians and vegans are significantly more in mismatch with their personal attitudes higher than the perceived social norm, compared to non-veg*ns. These results reflect previous research on minorities and how they differ from other members of the group (Hassouneh et al., 2014; Gutmann-Kahn and Lindstrom, 2015): being part of a minority does, indeed, accentuate the belief that one’s personal attitude is different from the social norm. It does so not only by polarizing minorities’ attitudes (i.e., in our sample veg*ns have stronger attitudes than others) but also by changing group members’ perception of social norms (i.e., veg*ns perceived the social norm to be less in favour of eating less meat than omnivores). This confirms the biased perception of the social norm found in previous studies: group members do not estimate accurately the actual social norm (Prentice and Miller, 1993; Guimond et al., 2015; Geiger and Swim, 2016). Our results, however, go one step further by showing that different group members do not necessarily misestimate the social norm in the same way.

The results also confirm the mismatch models’ effect on individual and group level intentions: self-other mismatch significantly explains vegetarians’ intentions to continue to act on an individual level and to participate in group actions. First, vegetarians continuing to have high individual intentions are, indeed, linked to the difference in personal opinion compared to other group members, and their higher levels of willingness to participate in normative change. These results are consistent with those found in previous studies (Falomir-Pichastor et al., 2008; Lalot et al., 2018) and can effectively explain how minorities fight the pressure to conform to social norms. Second, while the specific indirect effect “diet—mismatch—group level intentions” was nonsignificant, the total direct and indirect effects of the model were significant: being part of a minority accentuates intentions to act on a group-level (participating in demonstrations and voting for a green political party). This effect is significantly mediated by participants’ mismatch perception and their willingness to participate in normative change. The model, therefore, does not only explain how minorities maintain their original pro-environmental action: consistent with previous literature (Lalot et al., 2018) it can also motivate people to act pro-environmentally in ways they were not necessarily doing before.

### Theoretical and applied implications

These results have multiple theoretical and applied implications. From a theoretical perspective, this study shows the need to refine certain theories in social psychology. Indeed, the present study can first be used to nuance the current literature on normative influence. Previously, deviance was originally considered as behaviour that negatively impacts the group and that should be avoided (Sherif, 1936; Asch, 1956). So, conformism was thought to be the be-all and end-all for group members, when they were faced with a social norm. It is not, however, necessarily the case: when group members are exposed to a social norm, they can also be part of an active minority that expresses a deviating opinion with the aim of changing and bettering the group. Indeed, a panel of previous real-life events and studies show that social change does not happen by conforming to social norms: social change is more often than not only possible with the efforts of minority groups and isolated individuals (Mugny et al., 1983; Lalot et al., 2018). For example, Moscovici et al. (1969), Moscovici and Lage (1976), and Moscovici (1991) shows that minorities drive social change by expressing their non-conformist opinions and that they have a latent influence on others. Unlike majorities that cause temporary public attitudinal and behavioural change (i.e., “manifest influence”), minorities influence others more slowly and privately. With this latent influence, minorities inspire and gradually motivate other group members to change by exposing them to opinions and behaviours that are different and non-conformist (Bolderdijk and Jans, 2021; Nardini et al., 2021). This dissident behaviour can, therefore, have a positive effect on society and is what some researchers call constructive deviance (Packer, 2008; Galperin, 2012; Vadera et al., 2013). This study contributes to this line of research firstly by confirming that certain group members do practice dissident behaviours, and secondly by showing that minorities actively participate in non-conformist actions in the specific aims to provoke social change. Social change is not an unwanted consequence of their actions but seems to actually be part of their motivation to act.

Of course, even if we cannot claim why people are vegetarian (indeed, multiple factors can be involved in eating preferences, see Symmank et al., 2017 for a review), our results suggest that vegetarians having high individual and group level intentions can be partly explained by their heightened “self-other” mismatch and their higher willingness to change the social norm. It seems like they manage to maintain their intentions because they perceive a bigger self-other gap and they want to change the social norm, compared to non-vegetarians. As a logical part of future research, motivation to be vegetarian and to encourage others to reduce their meat consumption should be examined more deeply. For example, future research could extend these results by seeing whether vegetarians continue their meat-free diet once the social change has been achieved: would they maintain their polarized, almost extreme level, of pro-environmental intentions once most group members are doing their part to help the planet? Or would they no longer feel the need to do so and shift to a less restrictive diet (i.e., eating meat at most once a day)?

Our results can secondly shed some light on limitations to the theory of planned behaviour that has been discussed in prior research. Recent studies show that attitudes do not have as much influence on intentions and behaviour as previously suggested and that a positive attitude does not always finish in actual behaviour. This is known as the attitude-behaviour gap (Aschemann-Witzel and Niebuhr Aagaard, 2014; Farjam et al., 2019). This can partly explain why a significant amount of studies and interventions (notably communication campaigns) have solely relied on majority normative influence to motivate pro-environmental behaviours (Bergquist and Nilsson, 2016; Niemiec et al., 2020; Salazar et al., 2021). While this attitude-behaviour gap does effectively exist, it can be reduced by several means, notably by considering moderating variables (Conner et al., 2002; Farjam et al., 2019). For example, Conner et al. (2002) show that the attitude-behaviour gap is reduced when the individual’s attitude is not ambivalent. On a similar note, our results also show that individuals’ attitudes can have a more significant role in motivating pro-environmental behaviour when they do not match with the actual norm. These results, therefore, also join a second criticism brought forward concerning the theory of planned behaviour and the stipulated independence between personal attitudes and social norms. Indeed, models like the theory of reasoned action (Ajzen and Fishbein, 1977), further extended within the theory of planned behaviour (Ajzen, 1991), suggest that while social norms and personal attitudes can often predict intentions and behaviour, they always do so independently (Ajzen, 1988). The present study concludes, however, that considering the interactive effect between social norms and personal attitudes can improve the understanding of what motivates intentions and behaviours: if people’s personal attitudes do not match social norms, then their intentions are affected differently, compared to when they are matched. Including the interaction between social norms and personal attitudes significantly predicts intentions and therefore improves the understanding of what motivates intentions and behaviours.

Another theoretical question emerges from our results: would we find the same ‘attitude – social norms’ mismatch effect on intentions when taking into account the perceived descriptive or prescriptive characteristics of norms rather than the perceived general attitude of the group (i.e., the social norm in general)? Would a disagreement with an injunctive norm (what others believe is right/wrong) have a different effect than a disagreement with a descriptive norm (what others do)? Previous studies (Khamzina et al., 2021) show that different types of norms can effectively interact differently with personal attitudes. They find that perceived group norms (PGNs, which focus on attitudes of others) interact more with participants’ attitudes than subjective norms (norms that focus on ‘important’ others) do. This can be due to the fact that there is a bigger perception bias for perceived group norms than for subjective norms (Deffuant et al., 2022): people tend to perceive others’ far attitudes (PGN) further than they actually are and close attitudes closer to theirs than they really are. This is less the case for subjective norms. As subjective norms are conceptually closer to injunctive norms (see Thøgersen, 2006 for a review), the mismatch effect could also have a bigger effect when considering descriptive norms, rather than injunctive norms. Further studies empirically testing these hypotheses are, however, still needed in this area.

Finally, our results could be used during the creation of behavioural change interventions. Indeed, many studies have found a positive impact of interventions aimed at either changing the TPB variables and thus indirectly changing intentions and behaviour, or using these variables to directly change intentions and behaviours (Steinmetz et al., 2016). Ajzen and Schmidt (2020), therefore, recommend designing interventions that influence these variables because they could produce substantial changes in behaviour. When creating and testing future interventions aiming for behavioural change, researchers could use our results to nuance and adapt these interventions to target a larger sample of individuals (i.e., by accentuating the idea that people can lead normative change by following their more favourable attitudes). For example, using informational strategies (see Steg and Vlek, 2009, for a review on informational strategies) that broadcast normative messages could remind individuals that the social norm is less favourable than they would like, and consequently activate their willingness to change social norms. This would push them to participate more in environmental action (i.e., eat less meat, or participate in collective action). This intervention would, however, only heighten environmental behaviours for participants that perceive a mismatch with their attitudes higher than the norm.

### Limitations and future directions

Despite providing empirical support for the mismatch model and having important implications, this study presents some limitations—notably the experimental design. Since this study is cross-sectional, the pathways in the mediation analysis can only be considered correlational and not to be causal links. While this study is a first step toward explaining the mismatch effect on pro-environmental intentions and how minorities maintain their pro-environmental intentions, further studies need to be conducted with an experimental design capable of testing the causal pathways of this model (i.e., manipulating the self-other difference in pro-environmental attitudes). A second limitation concerns the sampling strategy. In this study, we aimed to test the mismatch model presented in the introduction by comparing people who already act pro-environmentally in real life (i.e., vegetarians and vegans by eating no meat) and those who do not yet (i.e., people who still eat meat). Comparing these two existing sub-groups in the French population offered initial evidence supporting the mismatch model as hypotheses are confirmed. Indeed, veg*ns had much higher scores on each mismatch variable. While we compare statistically balanced groups, our participants had to volunteer to take part in the research which may have been creating a self-selection sampling bias. Thus, one can argue that this could have led to participants with only certain characteristics wanting to participate in the study, and therefore not providing a representative sample for the study (see Sharma, 2017). This is a common bias for all the studies that use, as we did, volunteer procedures of recruitment and snowball sampling for the study of ‘hard-to-reach’ populations such as veg*ns. This limit should be considered when interpreting the presented results.

A third limit is that we focused on only one of the various behaviours that can be used to mitigate climate change (see Wynes and Nicholas, 2017). Indeed, we generally wanted to study behaviour that contributes to the reduction of greenhouse gas emissions, and reducing one’s meat consumption does so significantly (see Tukker and Jansen, 2006; Aubin, 2014). Further research could reinforce our results by conducting conceptual replications with other pro-environmental behaviours. If the mismatch model does effectively explain social change, a “self-other” difference should also explain people’s behavioural intentions in others areas where social change is also needed (using eco-transports rather than a car alone, reducing energy and water consumption for example). Moreover, we did not consider other important factors that could intervene and extend the understanding of individual dynamics that are involved in the motivation to participate in social change. For example, it could be interesting to examine the role of people’s perceptions of climate change because this could play an important role in whether they support climate policies, and act to mitigate and/or adapt to climate change. Indeed, people may hold different beliefs about the extent to which climate change is caused by humans and what consequences it will have, where, and when (Van Valkengoed et al., 2021). For example, believers should be more inclined to behave in a pro-environmental way, and even more so if they perceived that this is not the case for general others. Indeed, as they should perceive the highest urgency to react against climate change, they also should be more motivated to change social norms with regard to pro-environmental behaviours. Other variables could also moderate the mismatch model. For example, depending on the person’s perceived behavioural control (PBC, Fife-Schaw et al., 2007; La Barbera and Ajzen, 2020) on the target behaviour, and the perceived electability of the political party (Abramowitz, 1989; Sandri and Seddone, 2015; Mildenberger and Tingley, 2017), their individual and group level intentions may not be the same. The classic hypothesis for PBC would be that perceiving low behavioural control would reduce the beneficial effect of a ‘self-other’ difference on individual pro-environmental intentions (see Ajzen and Schmidt, 2020 for a review). But a recent study conducted by Khamzina et al. (2021), see also Deffuant et al. (2022) shows that the mismatch effect only influences peoples’ behaviour with low PBC—as if high PBC sufficed in motivating action but when perceived control is low, other factors need to come into play. These results concur with other studies (Guagnano et al., 1995) that show that attitudes predict behaviour less when behaviour is easily feasible. As for the perceived electability of the political party, perceiving a political party as unlikely to be elected could reduce the beneficial effects of the mismatch effect on voting for a said political party. It might not, however, have an effect on other group-level actions (i.e., participating in demonstrations and protests). So, while this study’s main focus was the interactive effect between social norms and personal attitudes drawn from the theory of planned behaviour, future studies could include measures of perceived control and perceived electability to see how they specifically influence the mismatch model.

Finally, only pro-environmental intentions were measured and not actual pro-environmental behaviour. Despite their importance in predicting action (Armitage and Conner, 2001; Riebl et al., 2015), intentions cannot be fully equated with actual behaviour. Therefore, future research needs to assess the mismatch model by measuring, or even observing pro-environmental actions.

## Conclusion

This research contributes to the literature on social change and provides a better understanding of how vegetarians maintain their pro-environmental intentions, despite the social pressure to do otherwise. Indeed, our findings suggest that they perceive a gap between their attitude and the social norms, and this motivates them to change the current social norm. This willingness to participate in normative change is what then leads to vegetarians maintaining their individual intentions, and even having higher levels of group-level pro-environmental intentions. Future behavioural change interventions should, therefore, consider these results to better support active minorities and to also lead others into participating in normative change.

## Data availability statement

The raw data supporting the conclusions of this article will be made available by the authors, without undue reservation. Materials and data are available at OSF at: https://osf.io/unmc8.

## Ethics statement

All procedures performed were in accordance with the ethical standards of the Clermont Université Auvergne Research Ethics Committee (IRB-UCA REC) and with the 1964 Helsinki Declaration and its later amendments or comparable ethical standards. Ethical permission to conduct this research was granted by the IRB-UCA REC Protocol N° IRB00011540-2021-53. Written informed consent for participation was not required for this study in accordance with the national legislation and the institutional requirements.

## Author contributions

RH, AN, KK, SG, SM, and MS: conceptualization. RH and AN: material development, formal analysis, original draft, and writing – review and editing. RH and SM: participant recruitment and ethical standards. KK, SM, SG, and MS: review and editing. AN: funding acquisition. All authors contributed to the article and approved the submitted version.

## Footnotes

1 This path became nonsignificant when we included covariates in the model: *b* = –0.13, *SE* = 0.19, *t* = –0.68, *p* = 0.49, 95%CI [–0.51; 0.25]). Other unstandardized Betas slightly changed due to the addition of parameters in the model, but no large fluctuation toward p-values was observed. In other words, with the exception of path c’ in model B which became nonsignificant, all other paths, for both models A and B are broadly the same as those described here.
