# Supplementary material for: Understanding individual and collective response to climate change: The role of a self-other mismatch
Source: Front Psychol. 2022 Sep 29;13:935209. doi: 10.3389/fpsyg.2022.935209 (PMC9558113; doi:10.3389/fpsyg.2022.935209)
Supplement: Supplementary file 1 [file Data_Sheet_1.PDF]

Supplementary Material

1. Sample details

The study was programmed with Qualtrics survey software. The participants were contacted by the researchers to take part in an online study on global warming and meat consumption. The message was shared on a variety of Facebook groups, either linked to vegetarian cooking, solidarity between inhabitants of different French towns, or groups made for the purpose of sharing surveys and we thus proceeded through snowball sampling. This online study was conducted from 26 July to 9 September 2021. A total of 787 persons clicked on the online study's link. We removed 258 people who didn't complete the study (32,78% of missing values) from the analyses. Additionally, and according to pre-established criteria, we removed 27 participants because they have not lived in France for more than one year. 2 more participants were excluded based on suspicious demographical answers (ie., differences in reporting gender at the beginning and the end of the study) and one because they were a minor (17 years-old). We therefore had a total of 499 participants (M_{age} = 34.83, SD_{age} = 12.86; *range* 18-76), of which 107 were men (21.4%), 387 women (77.6%) and 5 (1%) who either fit into a different category or preferred not to say. Most of the participants of this sample are French (*N* = 485; 97.2%) and have a university education (*N* = 482; 96.6%). 246 (49.3%) of said participants were vegetarians or vegan and the other 253 (50.7%) still eat meat. After having explored responses of participants on our different measures, we excluded 13 of them because they were outliers on at least two of the three indicators we used to detect outliers (i.e., Malhalanobis distance, Cook's distance and leverage). The final sample comprised 486 participants (97.3% French native; 78% women, 21 % men, 1 % others) who ranged from 18 to 76 years old (M_{age} = 34.84, *SD* = 12.80). Most of the participants had a university education (96.5%, see Stable1). 240 participants (49.4 %) declared themselves as vegetarian or vegan and 246 (50.6%) still eat meat. About half of the participants evaluated personal norms first (N = 255; 52.5%) while the other half evaluated social norms first (*N* = 231; 47.5%). Comparing our samples data to data from the French national institute of statistics and economic studies (INSEE 2019, 2021), we can see that our sample is slightly younger, and is more highly educated than the national population. Women are also overrepresented in our sample.

Stable 1 (supplement). Level of education

Level of education	Counts	% of Total	Cumulative %
No education	1	0.2	0.2
Middle school (brevet etc.)	16	3.3	3.5
High school (baccalaureate etc.)	81	16.7	20.2
Bachelor's degree (2 – 3 years after the baccalaureate)	168	34.5	54.7
Master's degree (4 – 5 years after the baccalaureate)	202	41.6	96.3
PhD or higher (8 years after the baccalaureate)	18	3.7	100.0
Total	486	100	100

2. Descriptive (Stable 2)

Stable 2 (supplement). Means, standard deviations, reliabilities, skewness and kurtosis

Variables	M	SD	alpha/*r*	Skewness	Kurtosis
Demographic and dispositional variables					
Sex	0,80	0,42	-	-1,096	0,358
Age	34,84	12,80	-	0,855	-0,227
Education level	4,25	0,90	-	-0,550	-0,053
Pol. Ideology	3,94	1,59	-	0,142	0,221
French identity	6,58	2,05	-	-0,772	0,101
Activism	4,38	1,67	-	-0,330	-0,545
Variables of interest					
Diet	0,51	0,50	-	-0,025	-2,008
Personal attitude	7,24	2,05	.88	-1,318	1,173
Social norms	4,44	2,00	.90	0,276	-0,437
Mismatch	2,80	2,71	-	-0,111	-0,477
Willingness to change social norms	7,31	2,25	-	-1,335	0,959
Individual-level intention	7,43	1,92	.83	-1,434	1,457
Group-level intention	5,33	2,25	.409***	-0,185	-0,726

Note. N = 486. All scales ranging from (1) *strongly disagree* to (9) *strongly agree.* Sex (0 = male; 1=female; 2=other); Educational level (1 no education to 6 phd or higher); Pol. Ideology = political ideology extreme ranging from (1) *extreme left-wing* to (9) *extreme right-wing*. Diet (0 = Veg*ns; 1 = non-veg*ns); Mismatch (personal attitude minus social norms perception). *** $p < .001$.

3. Correlations among variables (Stable3)

Stable 3 (supplement). Pearson's correlations among variables.

	Variables	1	2	3	4	5	6	7	8	9	10	11	12
1	Sexe	1											
2	Age	,104*	1										
3	Education level	-.046	-.216**	1									
4	Pol. Ideology	-.010	.067	-.155**	1								
5	French identity	-.082	.021	.002	.309**	1							
6	Activism	.064	.112*	-.032	-.199**	-.023	1						
7	Diet	-.154**	.011	-.035	.261**	.170**	-.337**	1					
8	Personal attitude	.166**	-.067	.081	-.233**	-.088	.357**	-.536**	1				
9	Social norms	.022	-.019	.017	.081	,111*	-.036	.145**	.105*	1			
10	Mismatch	.109*	-.037	.049	-.236**	-.148**	.296**	-.512**	.678**	-.660**	1		
11	Willingness to change social norms	.213**	-.050	.040	-.227**	-.079	.390**	-.466**	.769**	.044	.548**	1	
12	Individual-level intention	.165**	-.038	.062	-.208**	-.059	.379**	-.460**	.756**	.033	.547**	.745**	1
13	Group-level intention	.046	-.002	.046	-.401**	-.089*	.520**	-.337**	.436**	-.003	.332**	.462**	.472**

Note. N = 486. All scales ranging from (1) *strongly disagree* to (9) *strongly agree.* Sex (0 = male; 1=female; 2=other); Educational level (1 no education to 6 phd or higher); Pol. Ideology = political ideology ranging from (1) *extreme left-wing* to (9) *extreme right-wing*. Diet (0 = Veg*ns; 1 = non-veg*ns); Mismatch = personal attitude minus social norms perception. * $p < .05$. ** $p < .01$.

4. Effect of attitude and social norms's order of presentation

In order to check for the order of presentation effects on our results we run several analyses on our main dependent measures – notably for the personal attitude and social norm questions.

We first conducted a 2 (Diet: veg*ns versus non-veg*ans) * 2 (Order: personal attitude firstly presented versus social norms firstly presented) * 2 (type of measures: attitude vs norm) mixed ANOVA with the last variable as a within participant factor. All reported effects are significant at $p < .001$ except where it is mentioned. The results show that including the order of presentation doesn't change our main results. Indeed, we still observed the main effect of type of measures $F(1, 482) = 719.08$, $\eta^2_p = .59$, yielding a significant difference between personal attitudes and perceived social norms. Overall, participants had a more favorable attitude toward the reduction of meat consumption as a mean to fight against global warming ($M = 7.24$, $SD = 2.05$) than they perceived other French had ($M = 4.44$, $SD = 2.00$). This main effect was qualified by a significant interaction with the diet that participants follow $F(1, 482) = 170.89$, $\eta^2_p = .26$. Veg*an participants were more positive toward the reduction of meat consumption as a mean to fight against global warming ($M = 8.35$, $SE = 0.11$) than non-veg*n participants (M = 6.16, SE = 0.11, $F(1, 482) = 192.32$, $\eta^2_p = .28$). Veg*an participants ($M = 4.18$, $SE = 0.13$) perceived slightly less than Non-veg*n participants ($M = 4.73$, $SE = 0.12$) that French people are favorable toward the reduction of meat consumption as a mean to fight against global warming ($F(1, 482) = 9.58$, $p = .002$, $\eta^2_p = .02$). Moreover, the perceived difference between attitude and social norms was greater for participants that follow a veg*an diet (mean difference = 4.17, $SE = 0.15$; $F(1, 482) = 781.96$, $\eta^2_p = .62$) compared with participants who still eat meat (mean difference = 1.44, $SE = 0.14$; $F(1, 482) = 96.10$, $\eta^2_p = .16$). Thus, H1 was confirmed: veg*ns of our sample were more strongly in mismatch, with their attitude higher than the perceived social norms, compared to participants who still eat meat regularly. Of interest here, there was a main effect of the presentation of the measures on participants' mean scores ($F(1, 482) = 7.92$, $p = .005$, $\eta^2_p = .016$) which was qualified by the type of measures ($F(1, 482) = 14.41$, $\eta^2_p = .03$). When the norms were measured first, the perceived difference between attitude and social norms was smaller (mean difference = 2.41, $SE = 0.15$; $F(1, 482) = 252.10$, $\eta^2_p = .34$) than when the attitude were measured first (mean difference = 3.20, $SE = 0.14$; $F(1, 482) = 493.68$, $\eta^2_p = .50$). Of importance, the interaction between the order of presentation of the measures and the diet the participants follow was not significant ($F < 1$). In addition, the two-way interaction was also not significant ($F < 1$). These results were robust both when excluding or including control variables (i.e., gender, political ideology, activism, and French identification).

We then run three independent 2 (Diet : veg*ns versus non veg*ns) * 2 (Order: personal attitude firstly presented versus social norms firstly presented) on respectively individual intentions to act against climate change, group intentions to act against climate change, and willingness to participate in normative change. For all the analyses, only the mains effects of diet were significant (see Stable 4): compared with non-veg*ns participants, veg*ns reported higher intention to act at an individual level against global warming in reducing their meat consumption ($F(1, 482) = 130.70$, $p = .001$ $\eta^2_p = .22$) and at group level against global warming, by supporting green political parties and participating in collective action in favor of the climate ($F(1, 482) = 61.56$, $p = .001$ $\eta^2_p = .11$). Note that these results support H2a and H2b. Vegans were also more willing to participate in normative change concerning meat consumption in France, compared to non-vegans ($F(1, 482) = 133.46$, $p = .001$, $\eta^2_p = .21$). All other effects were nonsignificant (i.e., for the main effect of order all $Fs < 3.56$; $ps > .06$; for the interaction all $Fs < 1$, $ps < .34$). Including control variables gives rise to a main effect of order on both individual ($F(1, 478) = 7.18$, $p = .008$, $\eta^2_p = .015$; $M_{normfirst} = 7.54$, $SD = 1.76$ vs. $M_{attitudefirst} = 7.33$, $SD = 2.06$) and group intention ($F(1, 478) = 4.72$, $p = .03$, $\eta^2_p = .01$; $M_{normfirst} = 5.37$, $SD = 2.19$ vs $M_{attitudefirst} = 5.30$, $SD = 2.30$), but not on willingness to participate in normative change ($F(1, 478) = 3.43$, $p = .065$

η^2_p = .007). When measure of perceived norm was presented first, individual and group intentions were slightly superior. There were no interaction effects (all *Fs* < 1, *p* >.476).

Stable 4 (supplement). Means and standard deviation for main measures as a function of the diet that participants follow

	Veg*ans (n=240)		Non veg*ans (n=246)	
Variables	*M*	*SD*	*M*	*SD*
Individual-level intention	8.33	1.10	6.56	2.14
Group-level intention	6.10	2.13	4.59	2.10
Willingness to participate in normative change	8.37	1.19	6.27	2.55

5. Extended description of the serial mediation models A and B

Hayes' (2014) PROCESS macro v4.0 (model 6) was used to test the serial mediation of the relationship between the participant's diet to participants intention to act pro-environmentally (see Sfigure 1). We hypothesized that veg*ns participants should be more highly in mismatch than non-veg*ns participants, which then heightens their willingness to change norms. As a consequence, the latter then increases intentions to act pro-environmentally at both individual (Model A) and group level (Model B). This approach allows the indirect effect passing sequentially through each of these mediators to be tested. Bootstrapping (5000 samples) was applied to obtain bias-corrected 95% confidence intervals of the indirect effects, with mediation occurring when the CIs do not cross zero (Hayes, 2014).

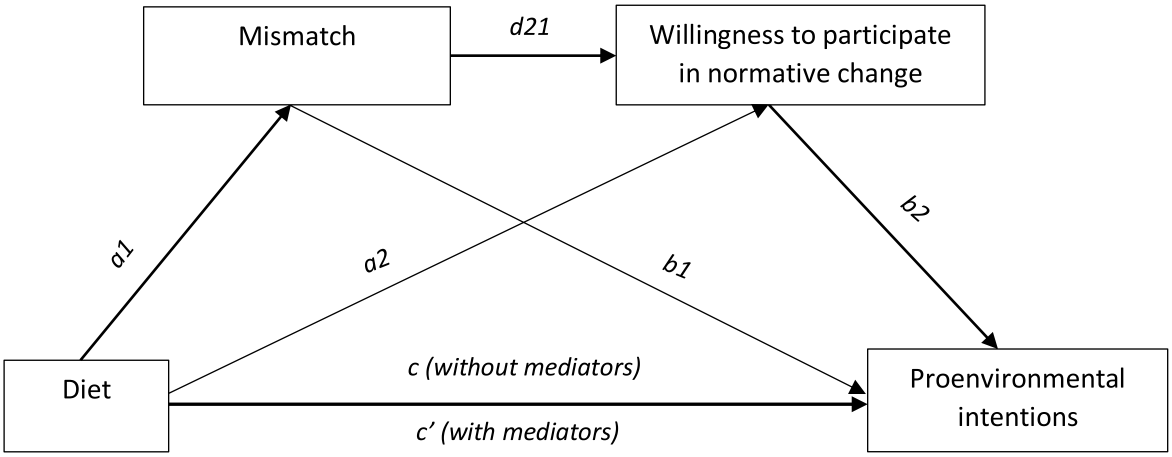

SFigure 1. Mediation model. Model A and model B were assessed using Hayes process model 6, evaluating through mismatch perception and willingness to participate in normative change as mediators of the relationship between the diet that participants followed (coded: 0 = Veg*ns; 1 = non-veg*ns); and the intentions to act against the climate change at an individual-level (Model A) and the diet that participants followed and the intentions to act against the climate change at a group-level respectively (Model B).

As predicted, it was found that participants' diet significantly predicted the mismatch perception (b = -2.77, SE = .21, t =-13.10, p < .001, 95%CI [-3.19; -2.36]; *path a1*), and explained 26% of variance in this variable (F(1,484) = 171.79, p < .001). This supported H1. Participants' diet (b = -1.13, SE = 0.19, t = -5.86, p < .001, 95%CI [-1.50; -.75]; *path a2*) and mismatch perception (b =.35, SE = 0.03, t = 9.81, p < .001, 95%CI [.28; .42]; *path d21)* also significantly predicted the willingness to participate in normative change. These predictors accounted for 34% of the willingness to participate in normative change, F(2,483) = 128.40, p < .001.

H2a and H2b were supported. Indeed, for the Model A and Model B, both the total effect of diet on intention to act at an individual or a group level against climate change (Model A: b = -1.76, *SE* = 0.15, t = -11.38, p < .001, 95%CI [-2.07; -1.46]; R^2 = .21; Model B: b = -1.51, SE = 0.19, t = -7.80, p <.001, 95%CI [-1.89; -1,13], R^2 = .11; *path c*) and the total direct effects when controlling for the mediators were significant (Model A: b = -.34, SE = 0.13, t = -2.55, p < .011, 95%CI [-.61; -.08]; and Model B: b = -.60, SE = 0.21, t = -2.81, p < .005, 95%CI [-1.03; -.18]; *path c'*).

Controlling for participant's diet and willingness to participate in normative change, the effect of mismatch perception on participant's intention to act against climate change at an individual level was significant (Model A: b = .11, *SE* = 0.02, t = 4.42, p < .001, 95%CI [.06; .17]; *path b1*), whereas it was not significant on participant's intention to act against climate change at a group level (Model B: b = .05, SE = 0.04, t = 1.22, p = .22, 95%CI [-.03; .13]; *path b1*). The willingness to participate in normative change significantly predicted, however, participant's intention to act against climate change at both an individual level (Model A: b = .52, SE = 0.03, t = 16.95, p < .001, 95%CI [.46; .58]; *path b2*) and a group-level (Model B: b = .36, SE = 0.05, t = 7.41, p < .001, 95%CI [.26; .46]; *path b2)*. Together, diet, mismatch and willingness to participate in normative change accounted for 59% of intention to act at an individual level (F(3,482) = 229.52, p < .001) and 23% at a group-level (F(3,482) = 49.39, p < .001).

Of importance, H3a and H3b were also supported. For both Model A and Model B, the total indirect effects were significant (Model A: *effect* = -1.42, bootSE = 0.14, boot95%CI [-1.70; -1.14], and Model B: *effect* = -.90, bootSE = 0.13, boot95%CI [-1.17; -.65]), with a significant serial mediation effect being observed from participant's diet via mismatch perception and willingness to participate in normative change to intention to act at an individual level (Model A : *effect* = -.50, bootSE = 0.08, boot95%CI [-.67; -.36]) and to intention to act against climate change at a group level *effect* = -.35, bootSE = 0.06, boot95%CI [-.49; -.23]). The specific indirect effect through mismatch only was significant for Model A (*effect* = -.32, bootSE = 0.08, boot95%CI [-.49; -.17]) but not for Model B (*effect* = -.14, bootSE = 0.12, boot95%CI [-.38; .10]) whereas the specific indirect effect through willingness to participate in normative change only was significant for both Model A (*effect* = -.59, bootSE = 0.10, boot95%CI [-.80; -.39]) and model B (*effect* = -.41, bootSE = 0.08, boot95%CI [-.59; -.26]).

Concerning our focal variables, results were robust both when excluding or including control variables for the two models. The only exception is that the total direct effect of participant's diet on group level intention (i.e., when controlling for the mediators) became non-significant [model B with covariates: b = -.13, SE = 0.19, t = -.68, p = .49, 95%CI [-.51; .25], path c'). Other unstandardized Betas slightly changed due to the addition of parameters in the model, but no large fluctuation toward p-values was observed. In other word, to the exception of path c' in model B which became nonsignificant, all other paths, for both model A and B are broadly the same than those describe here.

6. Additional mediation analysis on willingness to engage in normative change

To test whether the mismatch perception mediated the effect of the diet that participants follow on their willingness to engage in normative change, we used a bootstrapping procedure to compute the 95% CI around the indirect effect (i.e., the path through the mediator) using PROCESS macro in SPSS (Model 4, Hayes, 2014). Participants' diet (coded as 0 = Veg*ns; 1 = non-veg*ns) was entered along

with mismatch perception. Including covariates (i.e., counterbalancing, sex, political ideology, activism, and French identification) did not change our results. The path from participants' diet to mismatch perception was significant (*b* = -2.77, SE = 0.21, *t* =-13.10, *p* < .001, 95%CI [-3.19; -2.36]; *path a1*) and mismatch had a direct effect on willingness to participate in normative change (*b* =.35, SE = 0.03, *t* = 9.81, *p* < .001, 95%CI [.28; .42]; *path d21*). The significant effect of participant's diet was still significant when mismatch perception was controlled for (*b* = -1.13, SE = .19, *t* = -5.86, *p* < .001, 95%CI [-1.50; -.75]; *path a2*). However, results revealed that the indirect effect via mismatch equaled -.97, SE = .012, 95%CI [-1.23; -.74], suggesting a significant indirect effect of participant's diet on willingness to participate in normative change via the mismatch perception (mediation). Controlling for covariates doesn't change the results.
